# Cancer-Associated Fibroblasts as Players in Cancer Development and Progression and Their Role in Targeted Radionuclide Imaging and Therapy

**DOI:** 10.3390/cancers13051100

**Published:** 2021-03-04

**Authors:** Sofia Koustoulidou, Mark W. H. Hoorens, Simone U. Dalm, Shweta Mahajan, Reno Debets, Yann Seimbille, Marion de Jong

**Affiliations:** 1 Department of Radiology and Nuclear Medicine, Erasmus University Medical Center, Dr. Molewaterplein 40, 3015 GD Rotterdam, The Netherlands; m.hoorens@erasmusmc.nl (M.W.H.H.); s.dalm@erasmusmc.nl (S.U.D.); y.seimbille@erasmusmc.nl (Y.S.); m.hendriks-dejong@erasmusmc.nl (M.d.J.); 2 Department of Medical Oncology, Erasmus University Medical Center, Dr. Molewaterplein 40, 3015 GD Rotterdam, The Netherlands; j.debets@erasmusmc.nl (R.D.); s.mahajan@erasmusmc.nl (S.M.)

**Keywords:** CAFs, FAP, theranostics, radionuclide therapy

## Abstract

**Simple Summary:**

The tumour microenvironment contains a unique population of cells, of complex origin and diverse functionality, known as Cancer associated fibroblasts (CAFs). In recent years there has been a growing interest in targeting CAFs to aid cancer diagnosis and therapy. Amongst the approaches utilized so far, targeting Fibroblast activation protein (FAP) has shown a lot of promise. In this review, we will focus on our current knowledge of the biology of CAFs as well as theranostic applications that will enhance cancer diagnosis and therapy in cancers carrying a distinct CAF signature.

**Abstract:**

Cancer Associated Fibroblasts (CAFs) form a major component of the tumour microenvironment, they have a complex origin and execute diverse functions in tumour development and progression. As such, CAFs constitute an attractive target for novel therapeutic interventions that will aid both diagnosis and treatment of various cancers. There are, however, a few limitations in reaching successful translation of CAF targeted interventions from bench to bedside. Several approaches targeting CAFs have been investigated so far and a few CAF-targeting tracers have successfully been developed and applied. This includes tracers targeting Fibroblast Activation Protein (FAP) on CAFs. A number of FAP-targeting tracers have shown great promise in the clinic. In this review, we summarize our current knowledge of the functional heterogeneity and biology of CAFs in cancer. Moreover, we highlight the latest developments towards theranostic applications that will help tumour characterization, radioligand therapy and staging in cancers with a distinct CAF population.

## 1. Introduction

Cancer remains the second leading cause of death in the world [1], despite major improvements in diagnosis and therapy. This is partly because most of the developed therapeutics are targeting tumour cells ignoring however the surrounding tumour microenvironment (TME) [2]. The role of TME in cancer development has only gained attention in the recent years, driving research towards a new cancer treatment paradigm: the TME.

The TME may consist of more than 50% of the tumour mass and together with tumour cells it coordinates rapid proliferation, local invasion and eventually metastasis to the surrounding tissues [3]. In addition to cancer cells, the TME comprises immune cells, tumour vasculature, fibroblasts, extracellular matrix (ECM), myofibroblasts, neuroendocrine cells and adipose cells [4]. Intracellular communication is directed by a very complex network of cytokines, chemokines, inflammatory and matrix remodelling enzymes; the TME presents in fact similarities to the processes described in wound healing and inflammation.

TME-specific markers, found during tumour development, can be useful not only to understand the composition of TME, but also to aid diagnosis and therapy strategies [5]. It has been shown that spatial distribution of immune cells is positively correlated to patient survival in multiple tumour types (e.g., breast cancer and colorectal cancer) [6]. There are however, additional cell types within the TME that can regulate and affect oncogenesis. In particular, a subpopulation of fibroblasts called cancer associated fibroblasts (CAFs). These cells often constitute a major component in the TME, creating the ‘‘ground’’ in which the tumour cells can grow [7].

Growing evidence proves that CAFs can stimulate a malignant tumour phenotype due to their heterogeneous nature and functional diversity. Others suggest that a distinct CAF subpopulation, known as myofibroblasts (myCAFs), can have a more tumour-restraining role [8]. It is therefore, necessary to evaluate the importance of CAF biology and its various functions in cancer to identify specific subsets of the CAF populations that could constitute a springboard for more precise treatments. In this review, we aim to summarize the current understanding of the origin, and functional heterogeneity of CAFs in tumour development and progression. In addition, we outline potential strategies that target CAFs for their use in radionuclide imaging and therapy.

## 2. Cancer Associated Fibroblasts (CAFs)

Under normal conditions, fibroblasts are quiescent spindle shaped cells located in the connective tissue of most organs. They regulate the polarity and function of normal epithelium by generating the basement membrane. During tissue fibrosis and wound healing, these same fibroblasts are transformed into smooth muscle reactive fibroblasts and contribute to tissue repair [9]. The assumption that a tumour is a ‘wound that does not heal’ [10], has led to great interest in CAFs and their potential use in cancer therapy.

Studies have shown that CAFs are responsible for the synthesis and remodelling of the ECM and play various roles in cancer metastasis, angiogenesis, and drug resistance. Even though extensively studied in vitro, the CAF population remains largely uncharacterized in regards to its origin, biology, and subtypes [11], presenting phenotypic heterogeneity and functional diversity [12].

## 3. Origins of CAFs

**Figure 1:**
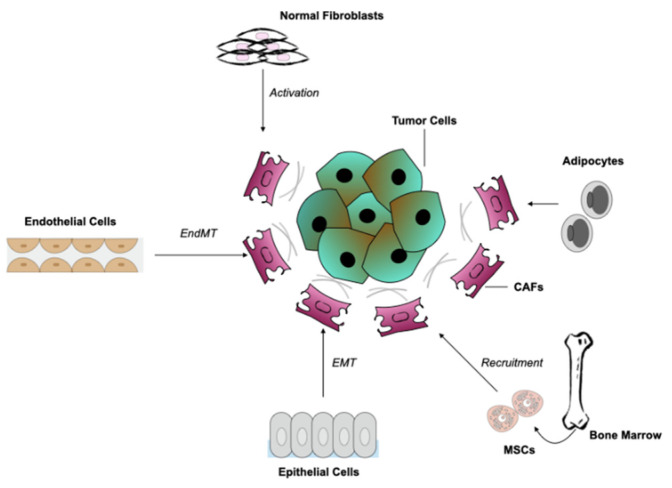
Potential cellular sources for cancer associated fibroblasts (CAFs). Several cellular types might yield CAFs in the TME. This includes normal fibroblasts through activation, recruitment and differentiation of bone marrow-derived mesenchymal stem cells (MSCs); epithelial cells through epithelial-to-mesenchymal transition (EMT) and endothelial cells through endothelial-to-mesenchymal transition (EndMT). Another source could be adipocytes through upregulated expression of mesenchymal bone marrow lineage-committed markers.

A number of different tissue types are responsible for the origin of CAFs (Figure 1), highlighting the heterogeneity of this specific cell population. CAFs can be produced from normal tissue fibroblasts that are activated by nearby tumour cells. This activation is strongly dependent on stimuli such as hypoxia, oxidative stress and growth factors produced by neighbouring tumour and immune cells [13]. More specifically, activation via the transforming growth factor-β (TGF-β), epidermal growth factor (EGF), platelet-derived growth factor (PDGF), and fibroblast growth factor 2 (FGF2), are the main contributors to fibroblast recruitment [14,15].

The best-studied cell population of CAF origin consist of mesenchymal stem cells (MSCs). In vitro and in vivo studies have provided evidence that MSCs from bone marrow (BM-MSCs) can differentiate into a CAF subtype in tumours such as gliomas, breast, and pancreatic cancers [16,17].

In addition to the adjacent sources, a number of CAFs can be identified from a non-fibroblastic origin. Such cells can be epithelial or endothelial cells, through epithelial-to-mesenchymal transition (EMT) or endothelial to mesenchymal transition (EndMT), respectively. The latter transition, was first observed during heart formation where an exchange in TGF-β signalling from endothelial to fibroblast-like cells was identified in cardiac tissues, suggesting that a similar phenomenon occurs in cancer tissues [18]. During this process loss of endothelial markers (e.g., CD31) and expression of mesenchymal markers (e.g., fibroblast specific protein (FSP) and α-smooth muscle actin (α-SMA)) is often visible.

Adipocytes can also transform into fibroblasts through expression of mesenchymal bone marrow lineage-committed markers (e.g., transcription factor (SOX9), peroxisome proliferator activated receptor-γ (PPARγ)) [15]. Adipocytes are located next to cancer cells and due to their interaction with tumour cells, they have become of major interest [19]. Moreover, an adipocyte-like cell population recruited from the bone marrow, known as fibrocytes, settles in the tumour stroma and is considered a subset of CAFs [20]. 

The difficulty of recognizing CAFs and their origin, lies in the absence of specific markers, although markers that are not expressed in normal cells can be used as an indicator, such as the aforementioned α-SMA [9]. Another way to distinguish them from other cells is by exclusion criteria based on their morphological characteristics (e.g., spindle shape characteristics) and the absence of non-mesenchymal markers [21]. In analysing tissue biopsies for example, cells not carrying epithelial, endothelial, and leukocyte markers, having elongated morphology and no mutations associated with cancer cells, can be considered CAFs. Taking into account the latter characteristic, cancer cells undergoing EMT can be also excluded. However, gaining a better understanding of the origin of this cell type and the various subsets could help to identify markers and ways to target these cells and their function.

## 4. Functions of CAFs

**Figure 2:**
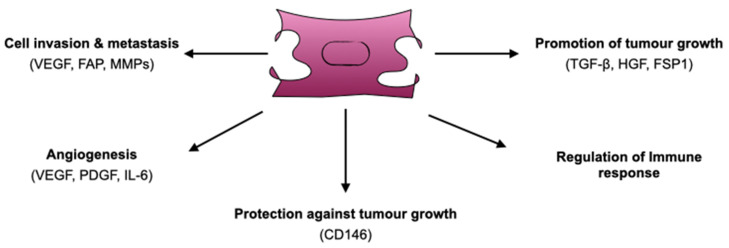
Diverse functions of CAFs: promotion of tumour growth, angiogenesis, cell invasion and metastasis to surrounding tissues, regulation of innate and adaptive immune responses. In addition to tumour-protective roles, CAFs occasionally have anti-tumorigenic functions. VEGF: Vascular Endothelial Growth Factor, FAP: Fibroblast Activation Protein, MMPs: Matrix Metalloproteinases, PDGF: Platelet-Derived Growth Factor, IL-6: Interleukin 6, CD146: Cluster of Differentiation 146, HGF: Hepatocyte Growth Factor, FSP1: Fibroblast-specific Protein 1.

Several studies utilizing cell culture as well as in vivo experiments in mouse models, have suggested a diverse number of functions that CAFs might employ. In their study, Olumi et al. were among the first to show that CAFs promote tumour growth in a prostate cancer mouse xenograft model while normal fibroblasts have more inhibitory properties [22]. In addition to tumour growth-promoting function of CAFs, the authors also described involvement in angiogenesis, tumour cell proliferation and metastasis, shedding light on the contribution of these cells towards malignant progression. In this section, we will refer to each of these functions separately (Figure 2).

### 4.1. Promotion of Tumour Growth

It is well known that tumour growth depends on the uncontrollable proliferation of tumour cells [23]. This rapid phenomenon can cause changes in the TME and besides stromal cells, CAFs have also been reported to cause induction of tumorigenesis through secretion of various growth factors and cytokines affecting the neighbouring cancer cells [24]. For example, overexpression of growth factor TGFβ and hepatocyte growth factor (HGF) in mouse fibroblasts was shown to induce breast cancer when co-injected with normal epithelium [25]. In another study, Grum-Schwensen and coworkers showed that Fibroblast-specific protein 1 (FSP1) knockout mice transplanted with cancer cells were less likely to form tumours when compared to mice injected with fibroblasts overexpressing FSP1. The evidence suggested that FSP1 secreted by CAFs alters the TME and thereby enhances cancer progression [26]. In a different example, expression of N-cadherin in melanoma cells allows interaction with N-cadherin-expressing fibroblasts to mediate cell adhesion and to improve the ability of melanoma cells to migrate through stroma and towards the vasculature. This expression of N-cadherin in fibroblasts during melanoma development not only provides adhesion ground to melanoma cells, but also promotes survival hence establishing a highly proliferative and malignant phenotype [27].

Reactive oxygen species (ROS) can modulate metabolic reprogramming of cancer cells but also of CAFs, thus supporting the acclimatization to oxidative stress that initiates CAF differentiation and tumorigenesis. Generation of ROS can trigger the initiation of events associated with metabolic switch in both cancer and CAF development. More specifically, production of hydrogen peroxide (H_2_O_2_) by cancer cells initiates oxidative stress in CAFs, followed by reduction of mitochondrial function, increase of glucose uptake and ROS levels, and finally leads to CAF differentiation. As a result, a reactive TME is generated by ROS, where CAFs sustain the energy needed for cancer cell proliferation [28,29]. Such environmental changes, and in particular increased H_2_O_2_ levels, can act as markers for therapeutic interventions [30].

### 4.2. Protection Against Tumour Growth

Notably, tumour-protective functions of CAFs have been also reported despite their potent role in tumorigenesis. In a mouse model of pancreatic ductal adenocarcinoma (PDAC), depletion of hedgehog signalling caused reduced stromal content, a more aggressive tumour phenotype and decreased survival [31]. The hypothesis was that the hedgehog-activated stroma prevents tumour angiogenesis and blocks tumour growth. In a different study, Brechbuhl et al. suggested that CAFs can affect oestrogen receptor (ER) expression and growth dependency in luminal breast cancer. In particular, breast cancer fibroblasts carrying a CD146-positive phenotype, may represent a subset of CAFs, which might confer oestrogen-dependent tumour cell proliferation and sensitivity to tamoxifen treatment [32].

### 4.3. Cell Invasion and Metastasis

In addition to promoting tumour growth, CAFs are also capable of rendering cells to invade surrounding tissues through cell-to-cell interactions and secretion of different invasive molecules (e.g., cytokines, chemokines, inflammatory molecules), hence, modifying the adjacent ECM [29]. The migration into the ECM is known to be a prerequisite for cancer cells to intravasate and ultimately metastasize [33]. In their study, Fukumura and coworkers used GFP transgenic mice under the control of the VEGF promoter and showed that CAFs positive for GFP were infiltrating tumour regions; however, it was difficult to assess whether it was CAFs contributing to this infiltration or the epithelial cells themselves [34]. One hypothesis was that CAFs may support the migration of cancer cells by guiding the migration rather than directly stimulating the cancer cells. In fact, in vitro co-culture experiments have suggested that cells of stromal origin are leading invasive cells by degrading the ECM through a combination of protease and force-mediated remodelling. Then, CAFs trigger structural modification of the ECM in order to pave the way towards the invasive cells [35]. In any case, it could be the cooperative interaction of these stromal cells with the epithelia that may lead to the invasive phenotype.

Enhanced expression and activation of matrix metalloproteinases (MMPs) by CAFs can also contribute to the remodelling of the ECM and thereby promote cell invasion. During normal homeostasis, MMPs keep the ECM in a balanced and well-organized state [36]. Overexpression of MMPs, however, can cause severe remodelling of the ECM, facilitating invasion and metastasis. In their study, Li et al. co-cultured primary CAFs with adenoid carcinoma cells (ACC) using a microfluidic device, where CAFs invaded the Matrigel via MMP activity. This could suggest that, besides the secretion of soluble factors, CAFs can create an invasive track in the ECM allowing other cells to follow them [37].

**Figure 3:**
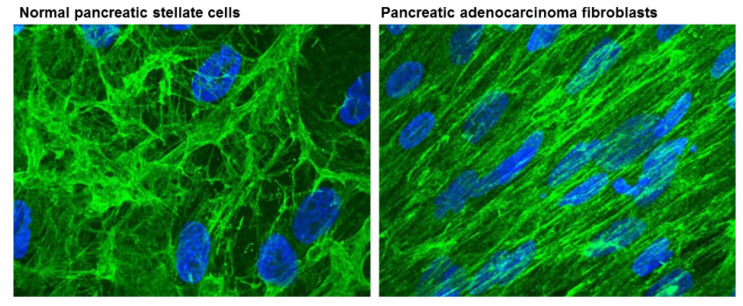
ECM fiber structures from normal pancreatic stellate cells and pancreatic adenocarcinoma associated fibroblasts stained for anti-fibronectin (green). ECMs organized in parallel patterns were observed in pancreatic adenocarcinoma fibroblasts that are similar to those formed by FAP-positive matrices. In contrast, ECM structures of normal pancreatic stellate cells resemble more FAP-negative matrices. Nuclei were stained with DAPI (blue). Reproduced with permission from [39].

Fibroblast activation protein (FAP), a type II membrane bound glycoprotein, can also contribute to matrix reorganization by acting as a serine protease to degrade type I collagen [38]. Lee et al. described that FAP acts through various proteins by modulating the protein levels, and also through increasing levels of fibronectin and collagen fiber organization. As such FAP promotes tumour invasion [39] (Figure 3). In a different study, Wang et al. reported that overexpression of FAP in a human hepatic stellate cell line caused cell migration via activation of ECM proteins, including MMP2 [40]. FAP expression levels have been found to correlate with poor disease prognosis in several types of cancer, including colon [41], pancreatic [42], and hepatocellular carcinoma [43]. Being the predominant component of cancer stroma in most types of cancer [38], FAP can therefore be considered a significant target for therapeutic intervention, which will be discussed in later sections.

### 4.4. Angiogenesis

Tumour growth and metastasis highly depend on angiogenesis for the tumour to develop, as the formation of new blood vessels is essential for the supply of oxygen and nutrients [44]. CAFs are capable to produce angiogenic factors, such as VEGF, and their production is induced by several components, the most important being hypoxia. In response to such conditions, CAFs upregulate the expression of VEGF protein in a manner that depends on oxygen deprivation [45]. CAFs can also affect angiogenesis through other angiogenic factors [46]. For example, the platelet derived growth factor/Platelet derived growth factor receptor (PDGF/PDGFR) signalling pathway has been described in angiogenesis through its involvement in CAF-mediated activation of VEGF. More specifically, PDGF directly attracts CAFs that secrete VEGF and thereby indirectly promotes angiogenesis [47].

Secretion of interleukin-6 (IL-6) by CAFs has also been reported to play a role in angiogenesis. IL-6 has the features of an angiogenic cytokine and was originally identified as a regulator of immune and inflammatory responses [48]. It is involved in various processes such as cell proliferation, migration, and angiogenesis. Dysregulated IL-6 production is associated with poor prognosis in many cancer types [49]. It was found that gastric cancer-derived isolated CAFs produced high amounts of IL-6 that enhance migration and EndMT of gastric cancer cells, a phenotype that was abrogated by inhibition of IL-6 [50]. These findings suggested that IL-6 can be directly inhibited to enhance responsiveness to therapy.

### 4.5. CAFs and Immune Response

It is becoming more and more evident that there is a cross-talk not only between tumour cells and immune cells, but also between CAFs and immune cells. This is because CAFs have been implied to affect both innate and adaptive immune responses [51].

Cells of the innate immune response (macrophages, neutrophils, dendritic cells (DC), natural killer cells (NK), mast cells, and cytotoxic T-lymphocytes) act as early responders to cancer-mediated inflammation, and also as precursors for the adaptive immunity [52]. The interaction between CAFs and M2 macrophages, carrying distinct signatures (e.g., high expression levels of FAP, α-SMA, and FSP1 for CAFs and expression of CD163 and Dendritic-Cell specific intercellular adhesion molecule-3-Grabbing Non-Integrin (DCSIGN) for macrophages), was reported in advanced colon cancer patients, and their co-presence was correlated with a differential disease progression and survival [53]. In particular high mRNA levels of CAF markers were correlated with poor disease-free survival and overall survival and when combined with M2 macrophage markers, these correlations became even more pronounced. In PDAC it was hypothesized that the communication between tumour cells and CAFs may be the initiator of cancer-associated inflammation [54]. This hypothesis was supported by the finding that the in vitro secretome of CAFs included immune cell chemokines (i.e., SDF-1) and anti-inflammatory proteins (i.e., CXCL6/GCP-2) that regulate the inflammatory response [54]. Pancreatic CAF precursors, known also as stellate cells, have been reported to interact with mast cells (e.g., a cell type that covers vessels and takes part in allergic responses) and tumour cells in models of PDAC. Together, tumour cells and stellate cells could activate mast cells in vitro, as was indicated by the mast cell release of TNF-α, thereby promoting tumour cell migration. Blocking this interaction led to tumour suppression [55].

With regards to cells of the adaptive immune system, the relationship between CAFs and the adaptive immune response is yet to be fully elucidated. This is in part due to the lack of sufficient and relevant multi-cellular in vitro assays as well as the majority of in vivo studies being performed in immune-suppressed animal models, both impeding the effects that CAFs can have on T cells. During early tumour initiation, naïve T cells are activated and migrate to the TME in order to eliminate cancer cells [56]. Findings from in vivo models suggest that when targeting specific CAF-related molecules, attenuation of tumour growth and metastasis will appear followed by a change of T cell response. For example, ablation of FAP-positive CAFs in vivo, in a breast cancer model, led to a switch from Th2- to Th1-type immunity (T cell mediated immune response is classified in Th1- or Th2-type immunity, based on the cytokine expression profile), followed by expression of cytotoxic cytokines IL-2 and IL-7 and increased CD8^+^ T cell tumour infiltration, all critical for tumour immunotherapy [57].

In 2013, Pena et al. reported that PDGF-activated fibroblasts could increase migration and invasion of colorectal cancer cells, a process driven by the glycoprotein stanniocalcin-1 (STC1) [58]. STC1 is known to exert anti-inflammatory responses that affect both the innate and adaptive immune system, either by affecting macrophage function through induction of UCP2 (e.g., reduces mitochondrial membrane potential necessary for proper immune response) [59], or via inhibiting the migration of both human macrophages and T-cells towards human umbilical vein endothelial cells (HUVEC) [60].

In short, these studies and others [61,62,63] suggest that during tumorigenesis, CAFs acquire the ability to recruit immune cells and later on modulate them to an immunosuppressive phenotype that is compatible with disease progression.

In light of the functional and morphological heterogeneity, one question that frequently appears is how does the transition of a normal fibroblast to CAF really occur? Studies suggest both genetic alterations [63,64] and epigenetic changes that may account responsible for this tumour-promoting phenotype, with the latest being the most prevalent [65,66,67,68,69].

### 4.6. The Role of miRNAs in CAFs

MicroRNAs (miRNA), which negatively regulate gene expression at a post-transcriptional level, have been reported to be involved in the conversion of fibroblasts to CAFs [69]. CAFs isolated from patients and analysed for miRNA expression presented 11 altered miRNAs when compared to normal fibroblasts [70]. Of those, three miRNAs (miR-221-5p, miR-31-3p, miR-221-3p) were found to be up-regulated, and eight (miR-205, miR-200b, miR-200c, miR-141, miR-101, miR-343-3p, let-7g, miR-26b) were down-regulated in CAFs. All 11 miRNAs affect important signalling pathways, such as those activated by IL-6, TGF-β, and hepatocyte growth factor, playing essential roles in cell proliferation, differentiation, cell migration, and interaction with TME components. It is noteworthy that CAFs could be transformed back to normal fibroblasts by mimicking this downregulation via gene transfer of miRNAs and miRNA inhibitors. On the other hand, normal fibroblasts containing reprogrammed miRNA showed upregulated expression of genes similar to CAFs. These genes were highly enriched in chemokines, known to be important in CAF function. This illustrates that miRNAs contribute to the cross-talk between CAFs and tumour cells. The aforementioned investigations provide strong evidence that epigenetic markers can be essential therapeutic targets for CAFs, and consequently for the TME.

## 5. Targeting CAFs as an Approach to Anti-Cancer Therapy

As mentioned earlier, a number of studies have suggested that CAFs and their actions are linked to disease outcome and therefore appear as a very attractive target for the development of anticancer therapies. In the past years, many have tried to target CAFs either directly by promoting conversion to a more normal fibroblast phenotype or indirectly by influencing their communication with nearby cells. Our increasing knowledge of CAF biology has led to more pre-clinical studies developing novel FAP-targeting anti-cancer strategies that could potentially show great promise in the clinic.

Several approaches have been applied on the targeting of CAF-related surface markers, such as FAP and α-SMA [71], since they are highly expressed in a number of tumour tissues, including pancreatic, lung, and breast cancer [72]. Some of these novel approaches include the use of monoclonal antibodies (mAb), small molecules [73], or chimeric antigen receptor (CAR) T-cells [74]; immunotherapy [75]; targeting of metabolism; conversion strategies.

### 5.1. Use of Antibodies, Small Molecules, or CAR-T Cells

The expression of FAP is unique for CAFs; FAP expression in other cells and tissues have been reported to be near undetectable [76]. This has led to the believe that chemical inhibition of FAP could have a therapeutic effect. In line with this, multiple FAP-targeting antibodies and smaller molecules have been developed and evaluated.

Ostermann et al. developed a promising mAb against FAP (FAP5-DM1), which showed long-lasting inhibition of tumour growth and good tolerability in preclinical mouse models of pancreatic, lung, and head-and-neck cancer [73]. Unfortunately, no tumour regression was observed during clinical trials in the majority of patients and the maximum tolerated dose was not reached with this mAb [77].

In 2013, Roberts et al. reported that depletion of FAP-positive cells from three sites (e.g., skeletal muscle, adipose tissue and pancreas) caused loss of muscle mass and reduction of erythropoiesis, and B-lymphopoiesis inducing cachexia and anaemia in mice [78]. This study strongly advocates that strategies targeting CAFs must consider the potential side effects that may appear under such a regime. Meanwhile, Feig and coworkers showed that depletion of FAP-positive CAFs, in PDAC-bearing mice, enabled the immune control of tumour growth and the antitumour activities of α-CTLA-4 and PDL1 antibodies [79].

Peptide-like small molecule inhibitors, such as Val-boro-Pro (also known as Talabostat or PT-100), were initially developed to target dipeptidyl peptidase IV (DPPIV) but also showed effect on FAP [80]. This inhibitor was tested in several clinical trials for the treatment of different types of cancer [81], yet never got approval for clinical use. It did manage however, to form the starting point for the development of FAP inhibitors with improved activity, better stability and higher selectivity [82,83,84]. Currently, the best available FAP inhibitor described in literature is UAMC-1110. UAMC-1110 has low nanomolar activity combined with more than 500-fold specificity for FAP over off-target peptidases such as DPPIV and PREP [85]. Currently, the effectiveness of UAMC-1110 and its analogues have been evaluated pre-clinically and to the best of our knowledge no reports of clinical evaluation are available.

In another study, Lo et al. demonstrated that depletion by adoptive transfer of FAP-targeted CAR T-cells caused immune independent effects on tumour growth through reduction of ECM components, either directly by disrupting stromal-cell and matrix-dependent signalling in tumour cells, or by inhibition of angiogenesis [74]. It was also reported that depletion of FAP-expressing cells in Lewis lung carcinoma tumour models triggered acute hypoxic death of both tumour cells and stroma cells, that was regulated by interferon-γ (IFN-γ) and tumour-necrosis factor-α (TNF-α). The authors suggested that such a strategy, in combination with other immune-therapeutics, may be more beneficial [86].

Furthermore, therapeutics currently entering clinical trials might be more efficient when combined with CAF-targeting approaches. Hirata et al. reported that melanoma cells carrying BRAF mutations are more tolerant to the BRAF inhibitor, PLX4720, in areas where dense stroma is present. The authors suggested that PLX4720 has an effect on tumour stroma through ECM remodelling and supplies signals that make tumour cells more tolerant to therapeutic interventions [87].

### 5.2. Blocking CAF Function with IL-6

Sun et al. showed for the first time that IL-6, produced by CAFs, supported degradation of oestrogen receptor-α (ER-α) via the ubiquitin-proteasome pathway. Upon administration of a proteasome inhibitor, sensitivity to chemotherapy was restored in breast cancer cells. The decreased expression of ER-α was suggested to be due to the induction of EMT by IL-6, and these effects disappeared by targeting CAFs with a IL-6 neutralizing antibody [88]. Since IL-6 is the main mediator of such effects, it could be targeted only in a portion of CAFs and therefore more precise markers are needed to efficiently discriminate IL-6-producing CAFs from the rest of the population.

### 5.3. Hijacking the Metabolic Needs

Tumour cells largely depend on glucose and glutamine for their metabolic needs and may hijack CAF metabolism in order to meet those requirements. This specific cross-talk between tumour cells and CAFs provides applications for anticancer therapy [89]. In prostate cancer, elevated glutamine levels were detected in CAFs, which correlated with activation of signalling pathways in tumour cells, most importantly RAS. As a result, the CAF-derived glutamine supports the mitochondrial metabolism and induces neuroendocrine differentiation and consequently resistance to androgen deprivation therapy (ADT) [90].

In PDAC, the pancreatic stellate cells constitute a major cell type of the tumour stroma. Upon activation, due to production of cytokines or oxidative stress, they abnormally proliferate and produce a large number of ECM components until they establish a myofibroblasts-like phenotype [91]. It is this phenotype that is responsible for the fibrotic environment observed in chronic pancreatitis and PDAC. In a study by Jacobetz et al., enzymatic breakdown of hyaluronic acid, a prominent component of the stroma environment, was shown to cause remodelling of microvasculature, leading to a more enhanced therapeutic response [92]. This highlighted a way of overcoming chemoresistance through impairment of the microenvironment.

### 5.4. Conversion of CAFs to a Quiescent Phenotype

Another approach that may potentially target CAFs is hidden in the regulatory pathways that cause the transformation of normal fibroblasts into CAFs [21]. In their extensive review paper, Melissary et al. provide a detailed overview of the currently known methods that fibroblasts use for their conversion into CAFs including synthetic activation, epigenetic and metabolic reprogramming [93]. Of those, the one most interesting is the Vitamin D receptor (VDR). VDR is expressed in the stroma environment of gastrointestinal tumours, acts as a modulator of CAFs and when targeted with the ligand calcipotriol, could reprogram CAFs to a more quiescent state, thereby reducing inflammation and pancreatitis [94]. However, the various functions of VDR still remain to be elucidated. In their recent study, Gorchs, et al. reported that even though pancreatic CAFs responded to therapeutic stimulation of VDR, by promoting a less tumour-supportive CAF phenotype, at the same time the T cell mediated response against tumour cells was less efficient [95]. This only raises questions about the suitability of such approach and maybe a combination with immunotherapy strategies can prove more beneficial.

**Figure 4:**
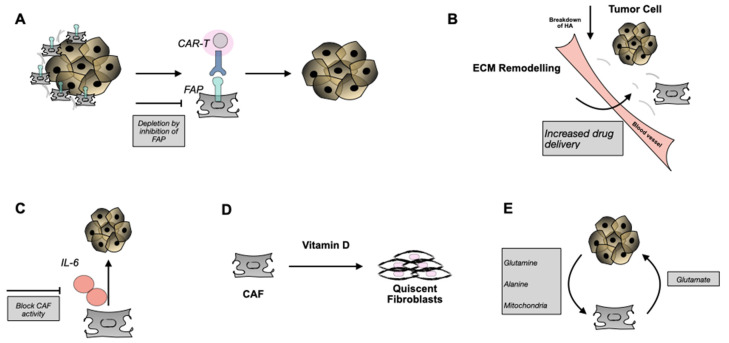
Overview of potential CAF interventions. (**A**) Use of anti-FAP mAbs or T-cells gene-engineered to express a FAP recognizing mAb (e.g., CAR-T cells) target FAP^+^ CAFs and result in their immune cell-mediated destruction and removal. (**B**) Enzymatic breakdown of HA may lead to remodelling of ECM and better tumour accessibility for drugs and/or immune cells. (**C**) Block CAF activity through IL-6. (**D**) Transform CAFs into a more quiescent state via Vitamin D. (**E**) Tackle the metabolic need of tumour cells and their dependence on CAF metabolism. FAP: fibroblast activated protein, CAR: chimeric antigen receptor, HA: hyaluronic acid, IL-6 interleukin-6.

The strategies described above (Figure 4) highlight the impact of targeting CAFs during treatment of cancer and also provide additional knowledge into their biology. Several approaches have been described and few are entering clinical testing and a number of them have been utilized to target CAFs with novel radiolabelled probes. The latter will be discussed in the following section. However, questions are still present that are related to the heterogeneity of this population and whether the same outcome will be observed in a clinical scenario. Careful analysis of the stromal responses will potentially aid our understanding of the source of CAF-mediated drug resistance, as well as spark a rational design for future patient studies targeting CAFs [10].

## 6. Targeting CAFs for Imaging and Therapy

As described above, the tumour stroma represents an attractive target for the delivery of diagnostic and therapeutic compounds. As mentioned earlier, CAFs feature high expression of FAP that is not detectable in adult normal tissue but is associated with a poor prognosis in cancer patients [41,42]. Several approaches have been applied to target CAFs with novel radiolabelled probes based on antibodies, peptides and small molecule inhibitors in different cancer types [72,96].

Fischer et al. [97] selected novel human Fab fragments from an antibody phage library that bound both human and murine FAP, two candidates were engineered into fully human IgG1 antibodies with affinities in the low nanomolar range. Radioimmunotherapy with ^177^Lu-labeled anti-FAP antibodies in melanoma-bearing mice delayed growth of established tumours and extended mouse survival, showing the potential for diagnostic and therapeutic use. No clinical studies have been performed yet with these tracers.

The development of the selective and potent FAP inhibitor UAMC-1110 has led to synthetization of other promising radiolabelled FAP inhibitors tested in different tumour entities (FAPIs) [98,99,100,101,102,103,104,105,106,107,108,109,110]. In particularly the quinoline group of UAMC-1110 allows for chemical modification: on the 5 and 6 position different chelators have been attached via various linkers. Lindner et al. [105] were the first to synthesize radiolabelled FAP inhibitors (FAPIs). Giesel et al. [98] described the tissue biodistribution and preliminary dosimetry of [^68^Ga]Ga-FAPI-2 and [^68^Ga]Ga-FAPI-4 in two patients, whereas further PET/CT scans were acquired of 25 patients. Similar to literature values for [^18^F]FDG (Average SUV_max_ 7.41, [98]), [^68^Ga]Ga-DOTA-TATE (Average SUV_max_ 16 ± 10.08, [111]), and [^68^Ga]Ga-PSMA-11 (Average SUV_max_ 11.3 ± 7.5, [112]) 200 MBq of [^68^Ga]Ga-FAPI-2 or [^68^Ga]Ga-FAPI-4 corresponded to an equivalent dose of approximately 3–4 mSv. Using [^68^Ga]Ga-FAPI-2, the tumour uptake from 1 to 3 h after injection decreased by 75%, whereas for [^68^Ga]Ga-FAPI-4 this was only 25%. In comparison to [^18^F]FDG at 1 h after injection, tumour uptake was almost equal. Continuing with [^68^Ga]-FAPI-4 PET/CT, Kratochwil et al. [103] quantified tumour uptake in eighty patients. The highest average SUVmax was found in sarcoma, oesophageal, breast, cholangiocarcinoma, and lung cancer, the lowest in pheochromocytoma, renal cell, differentiated thyroid, adenoid cystic, and gastric cancer, whereas the average SUVmax of hepatocellular, colorectal, head-neck, ovarian, pancreatic, and prostate cancer was intermediate. Because of low background in muscle and blood pool, tumour-to-background (TBR) contrast ratios were more than 3-fold in the intermediate and more than 6-fold in the high-intensity uptake group. FAP-specific PET imaging was also applied preclinically and clinically in gliomas [109]. IDH-wildtype glioblastomas and grade III/IV, but not grade II, IDH-mutant gliomas showed elevated tracer uptake. Immunohistochemistry showed FAP-positive cells in glioblastomas and an anaplastic IDH-mutant astrocytoma.

Next, several novel FAPI variants were described (Table 1). Tumour-to-normal-organ ratios were improved for most of the compounds, resulting in images with higher contrast, especially for FAPI-21 and -46 [106]. A separate study performed with [^68^Ga]Ga-FAPI-46 showed a favourable dosimetry profile with an estimated whole-body dose of 5.3 mSv for an administration of 200 MBq. The biodistribution study showed high TBRs increasing over time, making this an interesting tracer for future theranostic applications [108].

Loktev et al. [107] developed promising iodinated (FAPI-1) and DOTA-coupled (FAPI-2) radiotracers also based on FAPI. FAPI-1 showed time-dependent efflux and robust deiodination, whereas FAPI-2 showed enhanced binding and uptake to human FAP as compared with FAPI-01. [^99m^Tc]Tc-FAPIs were developed as well and showed specific binding to recombinant FAP-expressing cells with high affinity [104]. The lead candidate [^99m^Tc]Tc-FAPI-34 was applied for diagnostic scintigraphy and SPECT of patients with metastasized ovarian and pancreatic cancer for follow-up to therapy with [^90^Y]Y-FAPI-46. [^99m^Tc]Tc-FAPI-34 accumulated in the tumour lesions also shown in PET/CT imaging using [^68^Ga]Ga-FAPI-46, making this a powerful tracer for diagnostic scintigraphy, especially in cases where PET imaging is not available. Additionally, the chelator used in this compound allows labelling with the therapeutic radionuclide ^188^Re. Watabe et al. applied radionuclides with relatively long half-lives, ^64^Cu and ^225^Ac to label FAPIs for studies in mice with human pancreatic cancer xenografts. Accumulation levels in the tumour and most normal organs were significantly higher for [^64^Cu]Cu-FAPI-4 than for [^68^Ga]Ga-FAPI-4. [^225^Ac]Ac-FAPI-4 injection showed significant tumour growth suppression in PANC-1 xenograft mice indicating that [^64^Cu]Cu-FAPI-4 and [^225^Ac]Ac-FAPI-4 could be used in theranostics for the treatment of FAP-expressing pancreatic cancer [101].

Toms et al. [102] synthesized an ^18^F-labelled FAPI ([^18^F]FGlc-FAPI) and concluded from a preclinical study it is an interesting candidate for translation to the clinic, taking advantage of the longer half-life and physical imaging properties of ^18^F. Giesel et al. [113] describe the NOTA-chelator ligand FAPI-74 that can be labeled with both Al [^18^F]F and ^68^Ga. In ten patients with lung cancer PET-scans were acquired after administration of [^18^F]F-FAPI-74, with the highest contrast achieved 1 h p.i. in primary tumours, lymph node and distant metastases with SUVmax > 10, respectively. The radiation burden of a diagnostic [^18^F]F-FAPI-74 PET-scan was lower than that of PET-scans with [^18^F]F-FDG and other [^18^F]F-tracers, whereas [^68^Ga]Ga-FAPI-74 was comparable to other ^68^Ga-ligands. The high contrast and low radiation burden of FAPI-74 PET/CT favours multiple clinical applications. So, tumour-to-background contrast ratios of different FAPIs were equal to or even better than those of [^18^F]FDG [98,114]. In contrast to [^18^F]FDG, no diet or fasting in preparation for the examination is necessary, and image acquisition can potentially start a few minutes after tracer application. Despite their advantages, utilization of FAPIs in the clinic may face some limitations as there is still not enough information on its specificity, suggesting a decreased performance in patients who might have other diseases that are characterized by high levels of fibrosis.

The efficacy of clinical radioligand therapy via crossfire effects is not yet known. [^90^Y]Y-FAPI-4 was chosen for a proof of principle approach in a final stage breast cancer patient with bone metastases [105]. This was associated with a significant reduction of opioids given as pain medication. Furthermore, no side effects were observed, especially no therapy-related hematotoxicity.

## 7. Future Opportunities for FAP-Mediated Imaging and Treatment

The results of the first FAPI tracers are very encouraging for theranostic applications for a wide variety of tumours. Here, we highlight several opportunities for future FAP tracers.

As most clinically valuable tracers belong to one family of small molecule tracers, based on FAP inhibitor UAMC-1110, it is currently the only available FAP inhibitor with low nanomolar activity and high selectivity. This calls for the development of novel small molecule FAP inhibitors, of which the scaffolds can be used for the design of new FAP theranostics with new and potentially improved properties. Another strategy, adopted by Clovis Oncology, is based on a peptidomimetic (FAP-2286) to target FAP [115] and the much anticipated results will provide more clues on this method. The properties of FAP (its location on the outside of the CAF membrane and its unique expression) makes it an interesting target for peptide receptor radionuclide therapy (PRRT). The best example of PRRT is that of targeting the Somatostatin receptor in Neuroendocrine tumour patients [116]. This therapeutic strategy is even EMA and FDA approved. To the best of our knowledge, no FAP-targeted peptide-based molecule that can be applied for PRRT is reported in literature. However, development of such molecules, through for example phage-display screening platforms or chemical inactivation of FAP peptide substrates, will open new opportunities for FAP theranostics. A number of antibodies against FAP have been also developed and despite their superiority, in terms of affinity and specificity, such molecules can be challenging in regards to toxicity towards healthy tissues. Third, an opportunity for developing FAP theranostics are stimuli-responsive tracers, in which the enzymatic activity of FAP itself and the environment created by CAFs and tumour cells is employed. For example, site-specific enzymatic cleavage of FAP can result in the formation of nanoparticles [117] or release of functionality [118]. Furthermore, due to metabolic changes in CAFs and cancer cells [28], the TME is acidified harbouring increased levels of H_2_O_2_ and glutathione. These tumour specific properties are currently evaluated in pro-drug strategies [119,120,121] and could be of value in developing theranostic tracers.

## 8. Conclusions

Despite the increasing progress in cancer research and the development of novel therapeutics, conventional therapeutics are not adequate enough. The majority of efforts so far were only focused on targeting the cancer cells and even though highly abundant, CAFs have long been ignored in these efforts. The emerging role of CAFs is slowly appearing to be of great importance and their functions in tumour progression and metastasis have become an attractive target for imaging and therapy. However, a number of challenges need to be addressed in order to reach translation from bench to bedside. Looking at their structural and functional complexity, diagnostic markers that can specifically recognize CAFs are still limited. This complexity is also outlined by the various CAF cell origins. In the TME, CAFs co-exist with tumour cells providing them the paracrine niche and nutrients that are needed for tumours to grow. Breaking this contact and pinpointing the role of each of the microenvironment compartments, may appear pivotal for discoveries of novel therapeutic interventions. Fortunately, technological advances over the past years are opening new opportunities in understanding the complex biology of CAFs in cancer and will pave the way for new targets that can be used in future developments, one of those targets being FAP. The high and rather selective tumour uptake of very promising FAP-targeting tracers as described opens up applications for non-invasive tumour characterization, staging examinations, and radioligand therapy in many different cancers with a high content of activated fibroblasts.

## Figures and Tables

**Table 1:** FAP-targeting tracers used for theranostic applications.

Tracer	Chemical Structure	Radionuclides	Application	References
FAPI-2	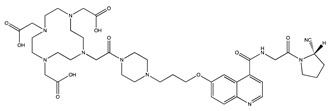	PET: ^68^Ga Therapy: ^177^Lu	Various Cancers	[98]
FAPI-4	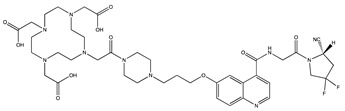	PET: ^68^Ga, ^64^Cu Therapy: ^90^Y, ^177^Lu, ^225^Ac	Breast Cancer	[105]
FAPI-21	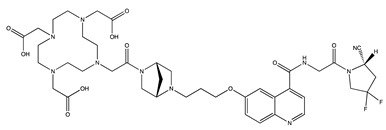	PET: ^68^Ga Therapy: ^177^Lu	Ovarian and Colorectal Cancer	[106]
FAPI-46	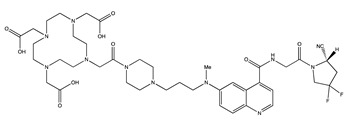	PET: ^68^Ga Therapy: ^90^Y, ^177^Lu	Colorectal, Mammary, Oropharyngeal Cancer	[106]
FAPI-34	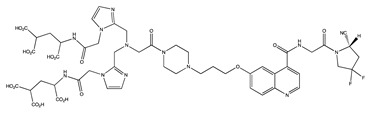	SPECT: ^99m^Tc Therapy: ^188^Re	Pancreatic, Ovarian Cancer	[104]
FAPI-74	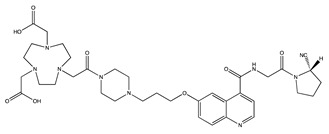	PET:^18^F, ^68^Ga	Lung Cancer	[113]
